# EARLY Treatment with azilsartan compared to ACE-inhibitors in anti-hypertensive therapy – rationale and design of the EARLY hypertension registry

**DOI:** 10.1186/1471-2261-13-46

**Published:** 2013-07-02

**Authors:** Anselm K Gitt, Peter Baumgart, Peter Bramlage, Felix Mahfoud, Sebastian A Potthoff, Jochen Senges, Steffen Schneider, Hartmut Buhck, Roland E Schmieder

**Affiliations:** 1 Stiftung Institut für Herzinfarktforschung, Bremser Strasse 79, 67063 Ludwigshafen, Germany; 2 Herzzentrum Ludwigshafen, Medizinische Klinik B, Ludwigshafen, Germany; 3 Clemens-Hospital Münster, Klinik für Innere Medizin I, Münster, Germany; 4 Institut für Pharmakologie und präventive Medizin, Mahlow, Germany; 5 Universitätsklinikum des Saarlandes, Klinik für Innere Medizin III, Homburg/Saar, Germany; 6 Universitätsklinikum Düsseldorf, Klinik für Nephrologie, Düsseldorf, Germany; 7 MedCommTools, medicalscientific consultancy, Hanover, Germany; 8 Universitätsklinikum Erlangen, Medizinische Klinik 4, Schwerpunkt Nephrologie / Hypertensiologie, Erlangen, Germany

**Keywords:** Azilsartan, Ramipril, ACE-Inhibitors, Non-Interventional, Blood Pressure, Follow-up, Ambulatory Blood Pressure Measurement, Central Systolic Blood Pressure

## Abstract

**Background:**

Arterial hypertension is highly prevalent but poorly controlled. Blood pressure (BP) reduction substantially reduces cardiovascular morbidity and mortality. Recent randomized, double-blind clinical trials demonstrated that azilsartan medoxomil (AZM) is more effective in reducing BP than the ubiquitary ACE inhibitor ramipril. Therefore, we aimed to test whether these can be verified under clinical practice conditions.

**Methods/Design:**

The “*Treatment with Azilsartan Compared to ACE-Inhibitors in Anti-Hypertensive Therapy*” (EARLY) registry is a prospective, observational, national, multicenter registry with a follow-up of up to 12 months. It will include up to 5000 patients on AZM or ACE-inhibitor monotherapy in a ratio of 7 to 3. A subgroup of patients will undergo 24-hour BP monitoring. EARLY has two co-primary objectives: 1) Description of the safety profile of azilsartan and 2) achievement of BP targets based on recent national and international guidelines for patients treated with azilsartan in comparison to those treated with ACE-inhibitors. The most important secondary endpoints are the determination of persistence with treatment and the documentation of cardiovascular and renal events. Recruitment commenced in January 2012 and will be completed by February 2013.

**Conclusions:**

The data obtained will supplement previous results from randomized controlled trials to document the potential value of utilizing azilsartan medoxomil in comparison to ACE-inhibitor treatment for target BP achievement in clinical practice.

## Background

Arterial hypertension is a highly prevalent disease, substantially impacting cardiovascular prognosis [1]. Despite the availability of many safe and effective antihypertensive drugs, of which angiotensin converting enzyme (ACE) inhibitors are the most widely used, blood pressure (BP) is controlled in only 20% of hypertensive patients [2]. An additional reduction of mean systolic BP in the order of 2 mmHg would result in a reduction of the rates of fatal stroke by 10% and of fatal myocardial infarction by 7% [1]. There is clearly a need for drugs that are potentially more effective and safe while having a high tolerability and persistence rates.

Azilsartan medoxomil (AZM, TAK-491) is a recently developed angiotensin receptor blocker (ARB) that has a number of specific characteristics [3]. It is structurally similar to candesartan except that it bears a 5-oxo-1,2,4-oxadiazolemoiety in place of the tetrazole ring [4]. Further it has a carboxyl group at the 7-position of the benzimidazole ring, which is believed to result in insurmountable receptor antagonism [3,5]. This insurmountable binding of AZM to the AT_1_ receptor may contribute to its potent and long-lasting antihypertensive activity.

### Azilsartan - clinical efficacy, safety and tolerability

Four studies compared the clinical efficacy of AZM with other ARBs. The studies included patients with primary hypertension and used ambulatory BP monitoring (ABPM) to determine BP lowering efficacy. AZM at a dose of 80 mg once daily provided superior BP lowering compared to the highest approved doses of olmesartan (40 mg), valsartan (320 mg) and candesartan (32 mg) [6-9] (Table 1).

**Table 1:** Clinical trials conducted with Azilsartan to date (Phase III)

	**Duration**	**Patients**	**Intervention**	**Primary endpoint**	**Outcome**
Sica et al. 2011 [7]	24	984 patients	AZM 40 mg/d (n=327)	Change in 24-hour	AZM 40 mg/d: -14.9 mmHg
		Mean age: 58 years	AZM 80 mg/d (n=329)	mean SBP from baseline to week 24	AZM 80 mg/d: -15.3 mmHg
		Female: 48%	Val 320 mg/d (n=328)		Val 320 mg/d: -11,3 mmHg
		BMI: 31 kg/m^2^			(p<0.001)
		Race: 77% Caucasian			
White et al. 2011 [6]	6	1291 patients	AZM 40 mg/d (n=280)	Change in 24-hour mean SBP from baseline to week 24	AZM 40 mg/d: -13.4 mmHg
		Mean age: 56 years	AZM 80 mg/d (n=285)		AZM 80 mg/d: -14.5 mmHg (p=0.009 vs. OLM, p<0.001 vs. VAL)
		Female: 46%	Valsartan 320 mg/d (n=282)		
		BMI: 31 kg/m^2^	Olmesartan 40 mg/d (n=290)		Valsartan 320 mg/d: -10.2 mmHg
		Race: 65% Caucasian	Placebo (n=154)		Olmesartan 40 mg/d: -12.0 mmHg
					Placebo: -0.3 mmHg
Bakris et al. 2011 [8]	6	1275 patients	AZM 20 mg/d (n=283)		AZM 20 mg/d: -12.2 mmHg
		Mean age: 50 years	AZM 40 mg/d (n=283)		AZM 40 mg/d: -13.5 mmHg
		Female: 50%	AZM 80 mg/d (n=285)		AZM 80 mg/d: -14.6 mmHg
		BMI: 30 kg/m^2^	OLM 40 mg/d (n=282)		(p=0.038 vs. OLM))
		Race: 73% Caucasian	Placebo (n=142)	Change in 24-hour mean SBP from baseline to week 6	OLM 40 mg/d:-12.6 mmHg Placebo: -1.4 mmHg
Rakugi et al. 2012 [9]	16	622 patients	Azilsartan up to 40 mg/d (n=313)	Change in sitting trough clinic DBP from baseline to week 16 (LOCF)	Azilsartan: -12.9 mmHg
		Mean age: 57 years	Candesartan up to 12 mg/d (n=309)		Candesartan: -9.7 mmHg
		Female: 39%			(p=0.0003)
		BMI: 25.5 kg/m^2^			(SBP reduction at week 16: AZM –
		Race: 100% Japanese			21.6 mmHg, CAN −17.3 mmHg)
Bönner et al. 2013 [11]	24	884 patients	AZM 40 mg/d (n=295)	Change in sitting trough clinic SBP from baseline to week 24	AZM 40 mg: -10,2 mmHg
		Mean age: 57 years	AZM 80 mg/d (n=294)		AZM 80 mg: -10,5 mmHg
		Female: 48%	Rami 10 mg/d (n=295)		Rami 10 mg: -4,9 mmHg
		BMI: 30 kg/m^2^			(clinic SBPp<0.001)
		Race: >99% Caucasian			

*Legend*: *LOCF*, Loss of Continous Follow-up; *SBP*, Systolic Blood Pressure; *BMI*, Body Mass Index; *CAN*, Candesartan.

Treatment was generally well-tolerated with a similar rate of adverse events between AZM and placebo and low rates of 2% to 3% for the withdrawal due to adverse events [10]. The most frequent adverse event was diarrhea in up to 2% of patients receiving the 80-mg dose compared with 0.5% of patients receiving placebo [10]. Other adverse events included nausea, asthenia, fatigue, muscle spasm, dizziness and cough. A small and reversible increase in serum creatinine was observed in patients receiving AZM 80 mg. Low hemoglobin, hematocrit, and red blood cell counts were observed only in 0.2%, 0.4%, and 0.3% of patients receiving AZM, respectively.

**Figure 1:**
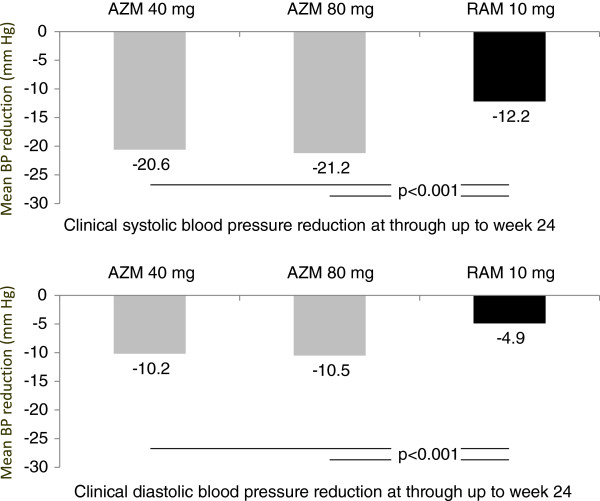
**Office blood pressure reduction in a comparative trial of Azilsartan Medoxomil (AZM) and ramipril (RAM)**[11]**.**

Recently, a randomized trial compared the antihypertensive efficacy and safety of AZM to ramipril [11]. This comparison is particularly important since ramipril is considered a benchmark antihypertensive drug that has not only been shown to be effective in lowering BP [12] but also to reduce cardiovascular morbidity and mortality in patients at high risk in the landmark Heart Outcomes Prevention Evaluation (HOPE) trial [13]. Patients in this comparative trial were assigned to receive daily doses of 40 mg AZM, 80 mg AZM or 10 mg ramipril. The primary efficacy endpoint was the change in clinic and ambulatory systolic BP from baseline. AZM 40 and 80 mg reduced both clinic and mean 24-hour systolic BP significantly more than ramipril at a dose of 10 mg did (clinic SBP −20.6 ± 0.9 with 40 mg and −21.2 ± 0.9 with 80 mg AZM vs. -12.2 ± 0.9 with ramipril) (Figure 1). Adverse events leading to discontinuation of treatment were less frequent with both doses of AZM (2.4% and 3.1%) compared with ramipril (4.8%).

### Aim of the registry

Based on these data, a considerable improvement of BP control might be expected from using AZM in clinical practice. The prospective observational registry (EARLY) aims at documenting BP control and its impact on cardiovascular and renal events during 12 months of follow-up in patients with either AZM or ACE-inhibitor treatment.

## Methods/Design

**Figure 2:**
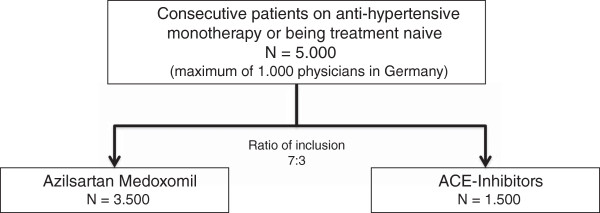
**Principal design of EARLY.** Patients will be recruited on a consecutive basis, given their compliance with the in- and exclusion criteria defined, in a ratio of 7 (AZM) to 3 (ACE inhibitors).

**Figure 3:**
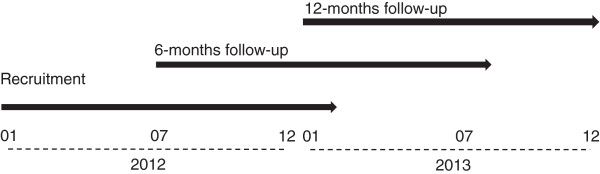
Patient recruitment and follow-up.

The “*Treatment with Azilsartan Compared to ACE-Inhibitors in Anti-Hypertensive Therapy*” (EARLY) registry is a prospective, observational, national, multicenter registry with a follow-up of 12 months. The registry will enroll up to 5000 patients with arterial hypertension starting treatment on either AZM or any ACE inhibitor monotherapy in up to 1000 sites in Germany in a ratio of 7 (AZM) to 3 (ACE-inhibitors) (Figure 2). While BP is regularly determined using office BP measurement, there is a subgroup of patients with ABPM using the Mobil-O-Graph® device. Data are recorded at baseline and will be prospectively documented during follow-up visits at 6 and 12 months or at the predefined end of the registry in December 2013 (Figure 3).

The registry is conducted in accordance with the ethical principles that have their origin in the Declaration of Helsinki and adheres to the principles of Good Epidemiology Practice (GEP), and applicable regulatory requirements. The protocol was approved by the independent ethics committee in Freiburg and the ethics committee of the State Medical Council of Rheinland-Pfalz, Germany. EARLY has further been registered with the database of the Verband forschender Arzneimittelhersteller (VFA). Patients being enrolled into this registry have to provide written informed consent.

### Objectives

EARLY has two **co-primary objectives:** 1) Description of the safety profile of AZM. 2) Achievement of BP targets based on recent national and international guidelines for patients treated with AZM in comparison to those treated with ACE-inhibitors.

**Secondary objectives** are as follows: 1) Absolute and relative BP reduction with antihypertensive treatment over the duration of one year. 2) Documentation of the adherence to guidelines for the diagnosis and treatment of hypertension in ambulatory care. 3) Persistence understood as the mean duration of monotherapy and / or AZM based combination therapy during follow-up. 4) Documentation of adverse events. 5) Prospective documentation of cardiovascular and renal events. 6) A pharmaco-economic evaluation of AZM use.

Additional objectives in a subgroup of patients with ABPM are to document BP lowering and pulse pressure reduction over the 1 year follow-up considering prior and concomitant antihypertensive pharmacotherapy as well as BP at baseline, and BP control with AZM in comparison to ACE-inhibitors (specific ACE inhibitors will be considered given their sample size is sufficient). Second, BP values obtained from ABPM will be compared to corresponding office BP values. Finally, central systolic and diastolic blood pressure, cardiac output (per minute), peripheral resistance, augmentation index, and pulse wave velocity will be determined and compared between patients receiving AZM or ACE-inhibitor treatment.

### Selection of sites

The registry is performed in primary care offices in Germany, with a planned participation of up to 1000 sites. Centers will be selected from a database maintained at the Institut für Herzinfarktforschung, Ludwigshafen, in order to be representative for the ambulatory treatment of hypertension in Germany. The recruitment per physician is limited to a maximum of 30 patients.

### Selection of patients

Adult patients (≥ 18 years) with essential arterial hypertension are included on a consecutive basis, given they have provide written informed consent and fulfill the following two criteria: 1) Participants have either no anti-hypertensive treatment prior to inclusion or a prior non-RAS based antihypertensive monotherapy. 2) A monotherapy using AZM or any ACE-inhibitor is initiated at baseline.

Patients are excluded from participation if they 1) receive antihypertensive drugs for an indication other than hypertension (e.g. beta blockers or diuretics for heart failure), 2) have a history of alcohol, drug abuse or illegal drug addiction, 3) have a life expectancy of less than one year, 4) are pregnant or breast feeding, or 5) are participating in other trials or registries. Further to this, patients with contraindications as to the summary of product characteristics of AZM or the ACE inhibitors will not be permitted. Patient recruitment started in January 2012 and will end on February 28^th^ 2013 at the latest or as soon as the recruitment target of 5000 patients is met.

### Documented variables

Table 2 displays the variables documented at baseline and at the follow-up visits at 6 and 12 months. These include socio-demographic variables, BP / heart rate, anamnestic concomitant disease, risk factors, anti-hypertensive treatment including dose, further pharmacotherapy, investigations, laboratory values, incident concomitant disease, lethal and non-lethal event rates and adverse events. In the subgroup of patients with ABPM respective parameters will be documented.

**Table 2:** Variables documented at baseline, and at 6 and 12 months of follow-up

	**Baseline**	**6 months FU**	**12 months FU**
Socio-demographic variables ^1^	X	X^9^	X^9^
Blood pressure / heart rate	X	X	X
Anamnestic concomitant disease ^2^	X		
Risk factors ^3^	X		
Anti-hypertensive treatment incl. dose	X	X	X
Further pharmacotherapy ^4^	X	X	X
Further investigations ^5^	X		
Laboratory values ^6^	X	X	X
ABPM ^7^ (subgroup)	X	X	X
Event rate (lethal / non-lethal)		X	X
Incident concomitant disease ^8^		X	X
Adverse events		X	X

Legend:

1) age, gender, height, weight, body mass index (computed).

2) heart failure (including NYHA class), coronary artery disease, stroke / transitory ischemic attack, atrial fibrillation, peripheral arterial disease, diabetes mellitus (including duration), kidney disease (including glomerular filtration rate <60 ml/1.73m^2^, dialysis or transplantation), malignancy, chronic obstructive pulmonary disease, depression and other severe diseases.

3) hypercholesterolemia, hyperlipidemia, smoking (present, past or never, including duration).

4) other than antihypertensive drugs: nitrates, platelet aggregation inhibitors, oral anticoagulants, statins or antidepressive drugs.

5) signs of left ventricular hypertrophy (ECG or Echo), intima / media thickness > 0.9 mm or plaque, ankle-brachial index (ABI) < 0.9, pulse wave velocity > 12 ms, microalbuminuria.

6) hemoglobin, glucose, bA1c, creatinine, sodium, potassium, calcium, phosphate, total cholesterol, LDL-C, HDL-C, triglycerides, C-reactive protein, uric acid, urinary albumin concentration and albumin to creatinine ratio (all if available within three months prior to visit.

7) mean blood pressure and heart rate during daytime, nighttime and 24-hour average.

8) corresponding to 2) including whether patients were hospitalized and for how long.

9) weight only.

### Quality assurance

There are three strategies for data quality checks: validations that occur at the time of data entry (i.e., “front-end”), a second, more sophisticated quality control program that runs as a prelude to the creation of the analysis data set and on-site data monitoring. Front-end data checks are advantageous because mistakes are caught and corrected at the time of entry – a system that is efficient for data collectors. Certain data elements can be required, while other variables may allow for missing values. Additionally, parameters will be defined to allow entry of only those records that meet inclusion criteria. Prior to the creation of the analytic dataset, more extensive quality control processes are performed. These checks, programmed in SAS, include parent–child edits, consistency edits, and data transformations that will facilitate analyses. Source documentation and data accuracy will be verified by site visits in randomly selected 5% of the sites.

### Data management and statistics

Data entry is performed by the physician or study nurse via a secure website directly into an electronic database. This approach allows online checks for plausibility and integrity.

The sample size for this registry was determined aiming to provide a sufficient precision of the primary endpoint, e.g. BP target achievement of <140/90 mmHg with a monotherapy of AZM or an ACE-inhibitor. Based on the aforementioned trial published by Bönner et al. [11] comparing AZM with the ACE-inhibitor ramipril, target achievement rates of 50-60% can be expected with AZM monotherapy versus 30-40% with ramipril. Because of the two principal research objectives each confidence interval (CI) is determined using the Bonferroni method for the adjusted confidence probability (1–0.05/2). For the AZM group a precision of the determined control rate of ±2.1% is planned (width of the 97.5% CI 4.2%), while a ±3.2% precision is deemed sufficient for the ACE-inhibitor arm. For this purpose 2849 patients have to be recruited for the AZM arm and 1178 patients for the ACE-inhibitor arm. Considering a design effect of 1.15 (because of the cluster-sampling design and allowing a drop-out of 5%), 3500 patients have to be recruited for the AZM and 1500 patients for the ACE-inhibitor arm, resulting in a total population of 5000 patients. There will be a proportional recruitment of patients until February 28^th^ 2013. In case the recruitment target of 5000 patients is not met, a reduced precision of the primary objective will be accepted.

All variables collected in the eCRF as well as the data obtained from the quality of life assessments and all derived parameters will be used in the statistical analysis. Binary, categorical, and ordinal parameters will be summarized by means of absolute and percentage numbers within the various categories (including ,,missing data‟ as valid category at baseline). Numerical data will be summarized by means of standard statistics (i.e. number of available data, number of missing data, mean, standard deviation, minimum, median, maximum, lower and upper quartile). In addition, adequate graphs (e.g. bar charts, box-whisker plots) may be presented to summarize the results for some parameters. Time-to-event variables will be analyzed via a Cox proportional hazard regression model presenting hazard ratios and the corresponding 95% confidence intervals (CI). In addition Kaplan-Meier curves will be presented for these variables. Two-sided 95%-CI will be presented for important parameters, but should be interpreted in an exploratory descriptive way. Further multivariable analyses will be performed according to the statistical analysis plan (SAP). Formal statistical tests will not be performed within the statistical analysis. A report including descriptive statistics of all documented parameters will be generated for the overall patient population. Depending on the variable(s) of interest, additional selection criteria for patients (e.g. subgroup analyses) considered in specific analyses may be used, if considered useful during the statistical analysis. Details on the selection criteria used will be given in the SAP and in the statistical section of the report. The statistical analysis will be performed using SAS (release 9.2 or higher; Cary, NC, USA).

## Discussion

### Value of non-interventional studies

Non-interventional studies supplement the results from randomized clinical trials (RCT) because they include patients that are frequently excluded from clinical trials because of stringent in- and exclusion criteria, thus representing clinical practice. This has been exemplified based on the *“Global Registry of Acute Coronary Events”* (GRACE registry) [14]. For this analysis 8469 patients were classified into three groups: included into contemporary RCTs (n=953), those eligible but not included (n=4669) and patients not eligible (n=2847). It was found that RCT participants had the lowest risk of death, eligible but not included had a higher risk and ineligible patients had the highest risk. Actual hospital mortality showed a similar gradient (3.6%, 7.1%, and 11.4%, respectively).

Similar observations were made in the recent non-interventional study SERVE [15]. Out of 8086 patients enrolled, only 2222 would have been eligible for the corresponding RCT COACH, based on the defined in- and exclusion criteria [16]. This led to an underestimation of the true BP lowering effect compared to the real life situation. It has to be acknowledged, however, that while the external validity of non-interventional studies is high, the internal validity is usually lower than in a RCT.

For the particular comparison of ARB with ACE-inhibitor treatment the EARLY registry is pursuing, available data is limited. This is mostly because non-interventional studies usually have no control group and are single-armed studies. If the results of a comparison are presented these mostly relate to differences between ARB treatment versus a stopped ACE-inhibitor at baseline. A more recent retrospective analysis by Roy and colleagues [17] used propensity score matching to balance two groups of patients receiving a new prescription of either ACE-inhibitors or ARBs on baseline factors. A total of 25035 patients were identified. No differences were found in the risk of death, coronary disease, chronic kidney disease, or stroke between those prescribed ACE-inhibitors or ARBs except for a higher rate of diabetes in patients treated with ARBs.

### Ambulatory BP monitoring substudy

There is a substudy in EARLY utilizing ABPM to verify and enhance diagnostic accuracy and add values not regularly obtained during office BP measurement. This is of particular importance since it has been shown that reductions in office-based BP values cannot to be translated 1:1 to 24-hour BP values. This was shown in a recent study in which lercanidipine was compared with enalapril in daily practice [18] using office (OBPM), ABPM and self measurement (SBPM). BP reductions derived from OBPM correlated to the values derived from SBPM and ABPM (day) at follow-up. However, correlation coefficients were usually low (range 0.05 -- 0.26) with highest coefficients for the correlation between SBP measured by OBPM and SBPM (r = 0.26). Lowest correlations were observed for OBPM and ABPM (r = 0.05 for SBP reductions). Higher values were seen in a further non-interventional study comparing OBPM and ABPM in patients receiving candesartan [19]. Correlation between OBPM and ABPM was substantially better with r = 0.589 for SBP and r = 0.389 for DBP during the day.

In addition to the brachial blood pressure measurements, the use of the mobilograph device allows to analyze the treatment induced changes of the central systolic blood pressure and pulse pressure as well as the augmentation index. Thus, we are able to examine the effects of AZM on central hemodynamic and vascular stiffness of large arteries.

### Limitations

There are a number of potential limitations to the design of the EARLY registry. Although we have a control group of patients receiving ACE-inhibitors, allowing to compare effectiveness and tolerability between AZM and ACE-inhibitors we have an 1) imbalance in group size, that will allow to determine control rates with AZM more precisely than in the control group. 2) The assignment to AZM or ACE-inhibitor treatment is performed by the treating physician and not the result of randomization. 3) Only a limited number of the available ACE inhibitors (e.g. ramipril) will be allowed because prescription rates of some of the other ACE inhibitors may be low. 4) Patients selected for AZM treatment may differ for known or unknown reasons from those receiving ACE inhibitor treatment. These limitations have to be weighed against the potential implications of EARLY findings: the registry includes a large group of unselected patients in primary care without the limitation given by stringent in- and exclusion criteria and has a 1 year follow-up that will allow to determine whether differences in BP lowering efficacy, persistence with treatment and tolerability might translate into a reduction of events throughout follow-up.

## Conclusions

The data obtained will supplement previous results from randomized controlled trials to document the potential value of utilizing azilsartan medoxomil in comparison to ACE inhibitor treatment for target BP achievement.

## Abbreviations

ABPM: Ambulatory blood pressure monitoring; ACE: Angiotensin converting enzyme; ARB: Angiotensin receptor blocker; AZM: Azilsartan medoxomil; BP: Blood pressure; CI: Confidence interval; DBP: Diastolic blood pressure; eCRF: Electronic case report form; GEP: Good epidemiology practice; OBPM: Office blood pressure measurement; SAP: Statistical analyses plan; SBP: Systolic blood pressure; SBPM: Self blood pressure measurement.

## Competing interests

Roland E. Schmieder (RES), Peter Baumgart (PBau), Peter Bramlage (PBra), Felix Mahfoud (FM), Sebastian A. Potthoff (SAP), Steffen Schneider (StS), and Anselm K. Gitt (AKG), have received research support and/or honoraria for lectures from a number of pharmaceutical companies producing anti-hypertensive drugs including Takeda the sponsor of this study. Hartmut Buhck (HB) is freelance consultant to the sponsor Takeda.

## Authors’ contributions

AKG, PBau, PBra, FM, SAP, JS, StS and RES have designed the registry. StS is responsible for the analysis of data. AKG, PB and HB drafted the manuscript based on the protocol and all other authors revised the article for important intellectual content. All authors have finally approved the version to be published.

## Authors’ information

EARLY Registry Group: Anselm K. Gitt (Co-Chair, Ludwigshafen), Peter Baumgart (Münster), Peter Bramlage (Mahlow), Felix Mahfoud (Homburg/Saar), Sebastian A. Potthoff (Düsseldorf), Jochen Senges (Ludwigshafen), Steffen Schneider (Ludwigshafen), Hartmut Buhck (Hanover), Roland E. Schmieder (Chair, Erlangen), Georg Lübben (previously Takeda), Claus Jünger (Ludwigshafen), Alexander Neumer (Ludwigshafen), Karin Vonderschmitt (Ludwigshafen), Reinhold Hübner (Berlin).

## Pre-publication history

The pre-publication history for this paper can be accessed here:

http://www.biomedcentral.com/1471-2261/13/46/prepub
